# Serum progranulin levels are elevated in patients with systemic lupus erythematosus, reflecting disease activity

**DOI:** 10.1186/ar4087

**Published:** 2012-11-11

**Authors:** Atsushi Tanaka, Hiroshi Tsukamoto, Hiroki Mitoma, Chikako Kiyohara, Naoyasu Ueda, Masahiro Ayano, Shun-ichiro Ohta, Yasushi Inoue, Yojirou Arinobu, Hiroaki Niiro, Takahiko Horiuchi, Koichi Akashi

**Affiliations:** 1 Department of Medicine and Biosystemic Science, Kyushu University, Graduate School of Medical Sciences, Fukuoka 812-8582, Japan; 2 Department of Internal Medicine, Saga University, 5-1-1 Nabeshima, Saga, 849-8501, Japan; 3 Research Fellowship Division Japan Society for the Promotion of Science, Sumitomo-Ichibancho FS Bldg., 8 Ichibancho, Chiyoda-ku, Tokyo 102-8472, Japan; 4 Department of Preventive Medicine, Kyushu University Graduate School of Medical Sciences, Fukuoka 812-8582, Japan; 5 Center for Cellular and Molecular Medicine, Kyushu University Hospital, Fukuoka 812-8582, Japan

## Abstract

**Introduction:**

Progranulin (PGRN) is the precursor of granulin (GRN), a soluble cofactor for toll-like receptor 9 (TLR9) signaling evoked by oligonucleotide (CpG)-DNA. Because TLR9 signaling plays an important role in systemic lupus erythematosus (SLE), we investigated whether PGRN is involved in the pathogenesis of SLE.

**Methods:**

We measured concentrations of serum PGRN and interleukin-6 (IL-6) with enzyme-linked immunosorbent assay (ELISA) in patients with SLE (*n *= 68) and in healthy controls (*n *= 60). We assessed the correlation between the serum PGRN levels and established disease-activity indexes. The sera from the patients with high PGRN titers (>80 ng/ml) at the initial evaluation were reevaluated after the disease was ameliorated by treatment. We also measured the IL-6 concentration secreted by peripheral blood mononuclear cells (PBMCs) incubated with (a) oligonucleotide (CpG-B) in the presence or absence of recombinant human PGRN (rhPGRN); and (b) lupus sera in the presence or absence of a neutralizing anti-PGRN antibody.

**Results:**

Serum PGRN levels were significantly higher in SLE patients than healthy controls. Their levels were significantly associated with activity of clinical symptoms. They also significantly correlated with values of clinical parameters, including the SLE Disease Activity Index and anti-double-stranded DNA antibody titers, and inversely with CH50, C3, and C4 levels. Moreover, serum PGRN levels significantly decreased after successful treatment of SLE. The rhPGRN significantly upregulated the production of IL-6 by PBMCs stimulated with CpG-B. Patients' sera stimulated production of IL-6 from PBMCs, which was significantly impaired by neutralization of PGRN. The serum PGRN levels significantly correlated with the serum IL-6 levels.

**Conclusions:**

Serum PGRN could be a useful biomarker for disease activity of SLE. PGRN may be involved in the pathogenesis of SLE partly by enhancing the TLR9 signaling.

## Introduction

Systemic lupus erythematosus (SLE) is a systemic autoimmune and inflammatory disease characterized by the polyclonal activation of T and B lymphocytes, production of autoantibodies, and formation of immune complexes that result in tissue and organ damage [1,2].

Toll-like receptor (TLR) signaling contributes to innate and adaptive immune responses [3]. TLR9 is a receptor for microbial CpG-DNA [4] and is expressed in plasmacytoid dendritic cells (pDCs), macrophages, and B-lymphocytes [5]. TLR9 recognizes unmethylated CpG oligonucleotides (CpG-ODNs), which are generally not present in mammalian cells [6]. However, in SLE, nucleic acid-containing autoantigens can be generated from apoptotic or necrotic cells [7] because of increased apoptosis, reduced clearance of apoptotic cells [8], and decreased methylation of DNA [9]. Patients with active SLE have increased TLR9 expression in peripheral blood memory and plasma B lymphocytes [10], and TLR9 signaling controls anti-DNA autoantibody production from these B cells in murine [11] and human lupus [12]. A genetic variation of TLR9 is associated with an increased risk of SLE [13]. These lines of evidence suggest that TLR9 signaling may play an important role in the pathogenesis of SLE.

Progranulin (PGRN; GenBank: NC_000017) is an extracellular glycoprotein, containing seven and a half repeats of cysteine-rich motifs. PGRN is proteolytically cleaved by extracellular proteases, such as proteinase 3 (PR3) and elastase, into granulin (GRN) [14], which ranges from 6 to 25 kDa. PGRN is abundantly expressed in rapidly cycling epithelial cells, leukocytes, chondrocytes, and neurons [15], and its expression level is at steady state [16]. PGRN plays a critical role in early embryogenesis [16], wound healing [17], maintenance of neuronal survival [18], and tumorigenesis [15]. Recent mouse studies show that mice unable to convert PGRN into GRN because of lack of both elastase and PR3 cannot show inflammation in response to injection of immune complexes [19]. These data indicate that PGRN is rapidly cleaved into GRNs in tissues by elastase to enhance inflammation. Moreover, GRN acts as a soluble cofactor for TLR9 signaling by binding to both CpG-ODNs and TLR9, thereby acting as a cross-linker for their interaction. GRN also promotes the delivery of CpG-ODNs to the endolysosomal compartments where TLR9 is localized [20].

Here, we show that serum PGRN levels are significantly elevated in SLE patients in parallel with disease activities and that PGRN may have a role in the pathogenesis of SLE partly by enhancing the TLR9 signaling and IL-6 production.

## Materials and methods

### Patients

We performed a cross-sectional study of patients who were treated for SLE at the Kyushu University hospital between the years of 2005 and 2011. In total, 68 Japanese patients with SLE were enrolled, and sera were obtained from these patients. All of the patients fulfilled at least four of the American College of Rheumatology (ACR) revised criteria for SLE. SLE disease activity was measured by using the SLE Disease Activity Index (SLEDAI) [21]. Active SLE was defined as a SLEDAI score ≥6 [21]. We excluded other autoimmune and infectious diseases. Each patient completed a standardized medical history, including drug use, and was given a physical examination. Serologic profiling of each patient was performed by using the standard immunoassays described later. The serum samples from the active SLE patients were acquired before the initiation or reinforcement of treatment, and the samples from the inactive SLE patients were acquired during regular hospital visits and then stored at -20°C.

The active SLE patients were treated with corticosteroids or immunosuppressive drugs after the completion of these evaluations. The sera obtained from the patients with high PGRN titers (>80 ng/ml) at the initial evaluation were reevaluated after the disease was ameliorated by treatment (*n *= 15). Control sera were obtained from healthy staff members (*n *= 60) in our hospital. This study was approved by the ethics committee of our institution, and the principles of the Helsinki Declaration were followed throughout the study. Informed consent was obtained from all participants.

### Data collection

The information obtained from the medical records of the patients included demographic data, such as age, sex, clinical manifestations of SLE, and laboratory values. Each SLE-related feature except anemia was defined according to the SLEDAI. Clinical features checked were malar rash/discoid rash, alopecia, oral or nasal ulcers, serositis, arthritis, active nephritis, CNS (central nervous system) lupus, vasculitis, fever >38°C, thrombocytopenia, leukopenia, and anemia. For example, thrombocytopenia was defined as a decrease in the number of platelets to <100,000/mm^3^. Leukopenia was defined as a decrease in the number of white blood cells to <3,000/mm^3^. Anemia was defined as a decrease in the concentration of hemoglobin to <10.0 g/dl for any cause. The data for the serum anti-Smith (anti-Sm), anti-ribonucleoprotein (anti-nRNP), anti-Ro/SS-A (anti-Ro), and anti-cardiolipin (anti-CL) antibodies were collected only from active SLE patients in whom these autoantibodies were measured (anti-Sm, *n *= 42; anti-nRNP, *n *= 35; anti-Ro, *n *= 39; and anti-CL, *n *= 44).

### The measurement of complement and autoantibody levels

The serum C3 and C4 levels were measured with turbidimetric immunoassay (Nittobo, Tokyo, Japan). The serum CH50 levels were measured with liposome immunosorbent assay (Wako, Tokyo, Japan). The serum anti-dsDNA, anti-Sm, anti-nRNP, and anti-Ro antibody levels were measured by using fluorescence-enzyme immunoassay (Phadia, Tokyo, Japan). The serum anti-CL antibody levels were measured with ELISA (MBL, Nagano, Japan).

### Immunoprecipitation

A primary antibody for PGRN (anti-PGRN Ab; R&D Systems, Minneapolis, MN, USA) or an isotype control antibody (control Ab; R&D Systems) was added to protein G-Sepharose (Pierce, Rockford, IL, USA) in phosphate-buffered saline (PBS) with 0.5% Triton-X and incubated on a shaker for 4 hours at 4°C. Recombinant human PGRN (rhPGRN; Cedarlane, Burlington, NC, USA) was added to the anti-PGRN Ab- or control Ab-protein G-Sepharose complexes and incubated on a shaker for 4 hours at 4°C. After washing the complexes, the proteins were eluted by boiling in sample buffer (0.125 *M *Tris-HCl, pH 6.8, 4% SDS, 20% glycerol, 3.1% dithiothreitol, and bromophenol blue). Proteins were separated by sodium dodecylsulfate-polyacrylamide gel electrophoresis (SDS-PAGE), transferred onto a nitrocellulose membrane, blocked with 5% skim milk in PBS with 0.1% Tween-20 for 1 hour, and probed with another anti-PGRN Ab (Epitomics, Burlingame, CA, USA) overnight at 4°C. The membranes were washed and incubated with horseradish peroxidase-conjugated streptavidin (Pierce) for 1 hour. The immunoblots were visualized with ECL detection reagent (Pierce).

### IL-6 induction

Peripheral blood mononuclear cells (PBMCs; 4 × 10^5 ^cells) from healthy control individuals were cultured with 250 ng/ml rhPGRN in serum-free medium (Invitrogen, Grand Island, NY, USA) for 2 hours to let PGRN partially convert into GRNs and were then stimulated with 10 n*M *CpG-B, 100 ng/ml poly (I:C) (agonist for TLR3) (InvivoGen, San Diego, CA, USA), 60 pg/ml LPS (agonist for TLR4) (Sigma-Aldrich, Saint Louis, MO, USA), or 1 µg/ml imiquimod (R837; agonist for TLR7) (InvivoGen). The PBMCs were also stimulated with 10% serum from SLE patients (*n *= 4) in the presence of anti-PGRN Ab or control Ab. In some experiments, lupus sera (*n *= 3) were treated for 1 hour with 6,000 U/ml DNase I (Roche, Penzberg, Germany) before stimulation. Triplicate cultures were grown in 96-well plates (Becton Dickinson, Franklin Lakes, NJ, USA) at a final volume of 200 µl/well. After 24 hours, the amount of IL-6 in the supernatant was measured.

### Measurement of PGRN, IL-6, IL-10, and immune complexes

The serum PGRN levels, serum and cell-culture supernatant IL-6 and IL-10 levels were determined by using ELISA kits (R&D Systems) according to the manufacturer's protocol. In brief, for PGRN, calibrators, control sera, and patients' sera (stored samples) were diluted and incubated with a mouse monoclonal antibody against PGRN, adsorbed onto the microtiter plate wells. After washing, a mouse monoclonal antibody against PGRN conjugated to horseradish peroxidase was added, followed by a second washing step and the addition of tetramethylbenzidine substrate. The intensity of the blue color developed was in proportion to the amount of PGRN bound in the initial step. The reaction was terminated by the addition of 2N sulfuric acid. The absorbance was measured in a microtiter plate reader (ThermoFisher Scientific, Waltham, MA, USA) and converted into nanograms per milliliter by plotting the values against the PGRN titer of the calibrators/standards given by the manufacturer. The assay range was 1.56 to 100 ng/ml.

Serum levels of immune complexes were measured with C1q solid-phase enzyme immunoassay according to the manufacturer's protocol (TFB, Tokyo, Japan).

### Statistical analysis

The differences between two groups were analyzed by using the Student *t *test. If there were a significant difference between the variances of the two samples, the *t *test corrected for unequal variances (the Welch *t *test) was applied. The Dunnett test is used for multiple comparisons with a control group. The relations between PGRN levels and other continuous variables were analyzed by using the Spearman rank correlation. The PGRN levels before and after treatment were compared by using a paired *t *test. The SLE patients were divided into three groups based on tertile distribution of the number of clinical features (0, 1 to 2, and 3 to 8). Linear trend across the tertiles was assessed by using ordinal variables coded 1 to 3. Among three groups, *P *values were calculated by analysis of variance and were adjusted by use of the Bonferroni correction. *P *values <0.05 were considered significant. All tests were two-tailed. All analyses were performed by using JMP statistical software (SAS Institute).

## Results

### The serum PGRN levels were elevated in patients with SLE

Of the 68 patients with SLE enrolled in the present study, 58 were women, and 10 were men (active, *n *= 46; inactive, *n *= 22). The patients ranged in age from 17 to 76 years (median age, 37 years). Meanwhile, in healthy controls, 51 were women, and nine were men. They ranged in age from 20 to 59 years (median age 32 years). No significant differences were found between patients with SLE and controls in terms of age and gender.

**Figure 1:**
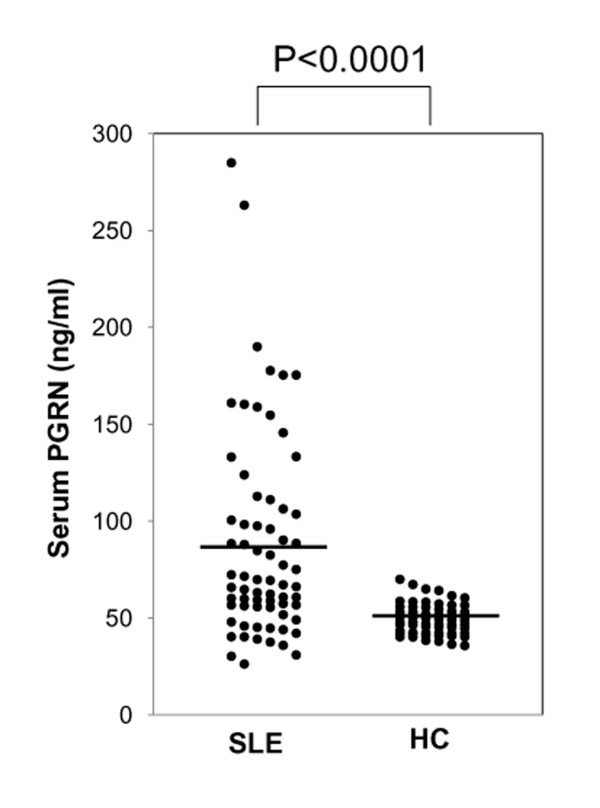
**The serum progranulin (PGRN) levels are elevated in patients with systemic lupus erythematosus (SLE)**. Serum PGRN levels in 68 patients with SLE and 60 healthy controls (HCs) are shown, as measured with enzyme-linked immunosorbent assay (ELISA). The serum PGRN levels in SLE patients were significantly higher than those in HCs.

To investigate the role of PGRN and/or GRN in the pathogenesis of SLE, we first compared serum PGRN levels between 68 patients with SLE and 60 healthy controls by using ELISA (Figure 1). Serum PGRN levels in controls were always within the range of 35 to 70 ng/ml and distributed normally. Serum PGRN levels in patients with SLE (mean, 87.6 ng/ml) were significantly and markedly higher than those in healthy controls (49.3 ng/ml; *P *< 0.0001). It is of note that serum PGRN levels >100 ng/ml were found in 19 of the 68 SLE patients (27.9%), but never in healthy controls.

### The serum PGRN levels in SLE patients correlated with disease activities

**Figure 2:**
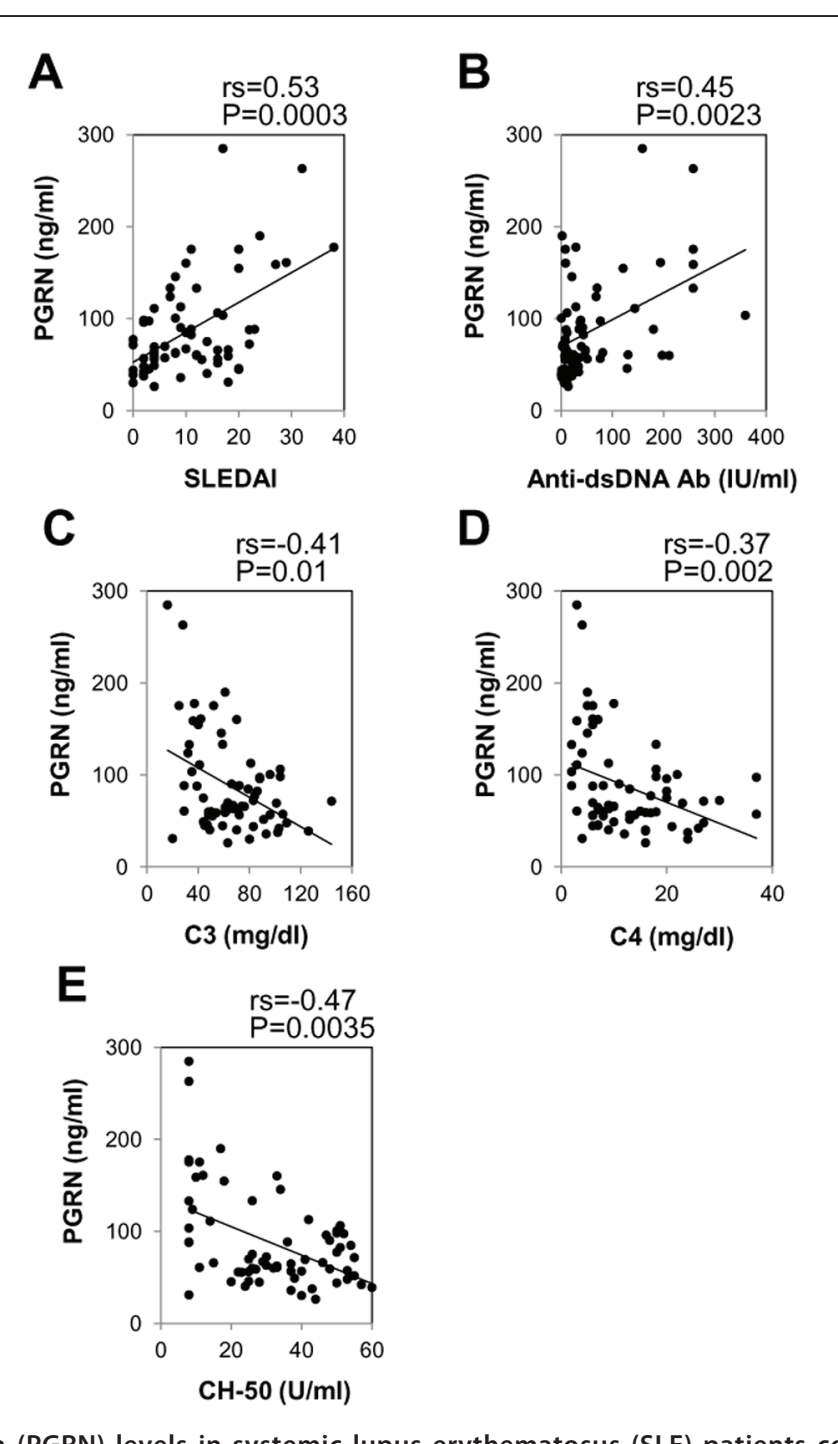
**The serum progranulin (PGRN) levels in systemic lupus erythematosus (SLE) patients correlate with disease activities**. Correlations are shown between the serum PGRN levels and the SLEDAI scores, the titers of **(A) **anti-dsDNA antibody (anti-dsDNA Ab) **(B)**, the serum levels of C3 **(C)**, C4 **(D)**, and CH50 **(E)**, as measured in 68 patients with SLE. For all comparisons, significant positive or negative correlations were observed.

**Figure 3:**
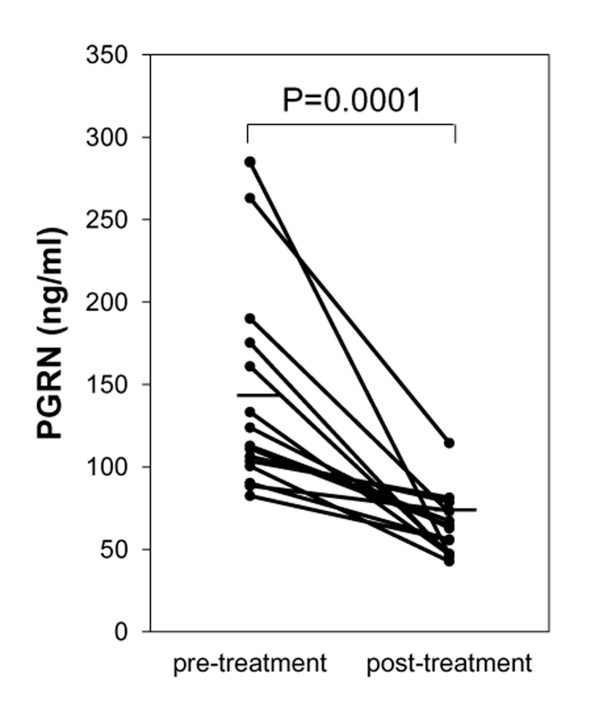
**Serum progranulin (PGRN) levels are significantly decreased after ameliorating the disease with treatment**. Serum PGRN levels before and after treatment in 15 patients with active systemic lupus erythematosus (SLE) who had serum PGRN levels >80 ng/ml at the initial evaluation. The serum PGRN levels decreased significantly with the clinical amelioration of the disease from the median SLEDAI score of 11 to 4 after treatment.

We next tested whether serum PGRN levels correlate with serologic parameters for disease activity of SLE. Serum PGRN levels showed a significantly positive correlation with SLEDAI (rs = 0.53; *P *= 0.0003; Figure 2A). They also significantly correlated with the titer of anti-dsDNA antibodies (rs = 0.45; *P *= 0.0023; Figure 2B) and inversely with serum levels of C3 (rs = -0.41; *P *= 0.01; Figure 2C), C4 (rs = -0.37; *P *= 0.002; Figure 2D), and CH50 (rs = -0.47; *P *= 0.0035; Figure 2E). Moreover, in 15 lupus patients who had high serum PGRN levels (>80 ng/ml) at the initial evaluation, after ameliorating the disease by treatment, serum PGRN levels were significantly decreased (*P *= 0.0001; Figure 3). Serum PGRN levels reflected disease activities of SLE.

### The high serum PGRN levels were associated with the accumulation of the clinical features of SLE

We next investigated the relation between SLE-related clinical features and serum PGRN levels. Patients who had positive levels of anti-nRNP antibodies showed significantly elevated levels of serum PGRN (*P *= 0.002; Table 1). The maximum number of coexisting clinical features was eight (*n *= 1). When classified by the number of clinical features, serum PGRN levels elevated significantly, with increasing tertile of clinical feature (Table 1, *P *for trend = 0.005). Thus, the high serum PGRN levels were associated with the accumulation of the clinical features of SLE.

**Table 1:** Associations between systemic lupus erythematosus (SLE)-related serologic and clinical features and titer of progranulin (PGRN)

Feature	Number	Mean PGRN (95% confidence interval)		*P*
			
		**Positive**^a^	**Negative**^a^	
Serologic feature^b^	Positive/negative			
Anti-Sm	25/17	111.8 (93.4-130.1)	94.1 (56.5-131.7)	0.381^c^
Anti-nRNP	23/12	128.9 (102.7-155.0)	74.9 (53.5-96.3)	0.002^c^
Anti-Ro	26/13	102.7 (79.6-125.8)	105.6 (67.6-143.6)	0.888
Anti-CL	20/24	112.4 (84.0-140.8)	87.4 (65.2-109.6)	0.150
				
Clinical features^d^				
0	22	51.3 (34.5-68.1)		<0.001^ef^
1-2	31	91.8 (81.1-102.5)		<0.001^e^
3-8	15	132.3 (113.1-151.5)		0.005^g^

anti-Sm, anti-Smith antibody; anti-nRNP, anti-ribonucleoprotein antibody; anti-Ro, anti-Ro/SS-A antibody; anti-CL, anti-cardiolipin antibody. ^a^SLE patients with (present) or without (absent) the clinical features of SLE. ^b^Several observations with missing values. ^c^Welch *t *test. ^d^Number of positive clinical features including malar rash/discoid rash, alopecia, oral or nasal ulcers, serositis, arthritis, active nephritis, CNS (central nervous system) lupus, vasculitis, temperature >38°C, thrombocytopenia, leukopenia, anemia. ^e^As compared with three to eight clinical features (one-way analysis of variance followed by the Bonferroni test). ^f^As compared with one to two clinical features (one-way analysis of variance followed by the Bonferroni test). ^g^*P *for trend.

### PGRN is involved in the production of IL-6 from human PBMCs

GRN enhances the CpG-induced TLR9 signaling (not TLR 3, 4, or 7 signaling) and promotes the production of IL-6, an inflammatory cytokine related to the pathogenesis of SLE, in murine macrophages [20]. Accordingly, we tested whether PGRN is involved in secretion of IL-6 via TLR9 signaling by human PBMCs. In the lupus model mouse, TLR 3, 4, 7, and 9 signaling are reported to be involved in the pathogenesis of lupus [22-24]. PBMCs from healthy controls were incubated with 10 n*M *(suboptimal concentration in our case; data not shown) CpG-B, 60 pg/ml LPS, 100 ng/ml poly (I:C), or 1 µg/ml imiquimod for 24 hours with or without 250 ng/ml rhPGRN. This rhPGRN concentration is slightly lower than the PGRN concentration we found in patients' sera (Figure 1).

**Figure 4:**
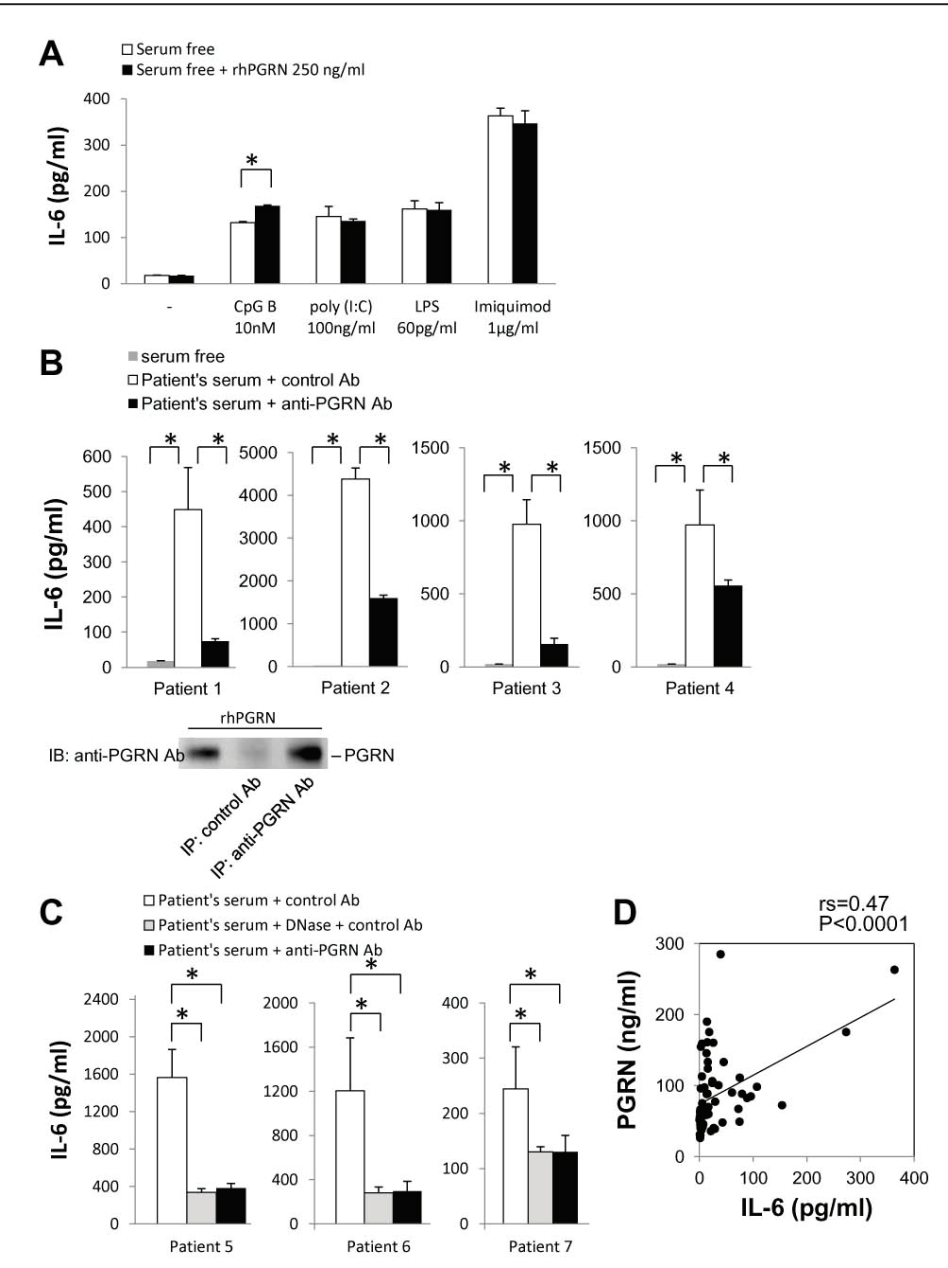
**Progranulin (PGRN) and/or its fragments GRNs augment the IL-6 production by human peripheral blood mononuclear cells (PBMCs) via TLR9 signaling**. **(A) **PBMCs from healthy control individuals were incubated for 24 hours in serum-free medium with or without 10 n*M *CpG-B, 100 ng/ml poly (I:C), 60 pg/ml LPS, or 1 µg/ml imiquimod and purified PGRN (250 ng/ml). The supernatant IL-6 levels were measured with ELISA. The data are representative of three independent experiments (average and SEM). **(B, C) **The binding of anti-PGRN Ab to PGRN was verified by immunoprecipitation and immunoblot with anti-PGRN Ab. In C, lupus sera were partly treated for 1 hour with 6,000 U/ml DNase I before stimulation. The PBMCs were then incubated in serum-free medium or were stimulated with 10% serum from systemic lupus erythematosus (SLE) patients in the presence of anti-PGRN Ab or control Ab for 24 hours. The supernatant IL-6 levels were measured with ELISA. **(D) **The serum PGRN levels correlated significantly with the serum IL-6 levels in SLE patients. *P < 0.05.

The addition of rhPGRN to the culture media significantly stimulated PBMCs to produce IL-6 in the presence of CpG-B (*P *< 0.05; Figure 4A). However, IL-6 production induced by rhPGRN was not augmented in the presence of poly (I:C), LPS, or imiquimod (Figure 4A), as previously reported [20]. Lupus serum contains immune complexes and is able to induce the production of IL-6 and IL-10 from PBMCs [25,26]. Actually, immune complexes were present in patients' sera we used (Patients 1 through 7, median, 6.1 µg/ml; range, 2.6 to 9.7 µg/ml). Those levels in healthy controls (*n *= 7) were less than 1.5 µg/ml. Consistent with the previous reports, by incubation with patients' sera, PBMCs from healthy controls were stimulated to produce considerable amounts of IL-6 and IL-10 (Figure 4B, and data not shown).

We then tested whether this stimulation of IL-6 and IL-10 production is dependent on the PGRN contained in patients' sera. To this end, we used an anti-PGRN antibody that can bind to PGRN demonstrated by immunoprecipitation and immunoblot analyses (Figure 4B, bottom). We incubated PBMCs with 10% patients' sera supplemented with the anti-PGRN Ab or with the control Ab for 24 hours. The neutralization of PGRN significantly impaired the effect of patients' sera on IL-6 production from normal PBMCs (*P *< 0.05) in all four cases tested (Figure 4B, top; reduction range, 42.8% to 84%). Although IL-10 production tended to be reduced by the neutralization of PGRN, the level of reduction was not significant (data not shown).

We then tested whether TLR9 signaling is involved in this reduction of IL-6 production by neutralizing the PGRN contained in patients' sera. To this end, we treated lupus sera (*n *= 3) with 6,000 U/ml DNase I for 1 hour before stimulation. The degradation of DNA significantly impaired the effect of patients' sera on IL-6 production from normal PBMCs. The degree of suppression was almost as same as that caused by the neutralization of PGRN (*P *< 0.05; Figure 4C).

We further examined the correlation between concentrations of PGRN and IL-6 in patients' sera. As expected, serum PGRN levels showed a significantly positive correlation with serum IL-6 levels (rs = 0.47; *P *< 0.0001; Figure 4D). These results suggest that serum PGRN and/or GRN is an important cofactor in the production of IL-6 in SLE patients.

## Discussion

This is the first study to show that PGRN levels are significantly elevated in sera of SLE patients, and their concentrations were correlated with disease activity and serum levels of IL-6. Thus, PGRN and/or GRN in patients' sera play an important role in the production of IL-6 from PBMCs.

PGRN is produced by various types of cells [15], but the mechanism of the expression control of PGRN is not well understood. In humans, the elevation of PGRN is observed in local inflammatory tissues, such as in brains in patients with active multiple sclerosis [27] and in the synovium of rheumatoid arthritis (RA) patients [28]. We measured concentrations of serum PGRN in patients with RA (*n *= 33). In our data, although sera from RA patients contained significantly elevated PGRN (mean, 54.7 ng/ml) compared with healthy controls (*P *= 0.0138), SLE patients' sera had much higher levels of PGRN.

PGRN is converted to GRN by the elastase produced by leukocytes and other cells *in vivo *and *in vitro *[29]. In contrast to that with PGRN, recent mouse studies have shown that GRN possess inflammatory functions [19,30,31]. Because we cannot measure serum GRNs, it is difficult to investigate the function of GRN in human studies.

Our data also show that rhPGRN enhances IL-6 production from human PBMCs in the presence of TLR9 signaling triggered by CpG, but does not enhance TLR3, 4, or 7 signaling (Figure 4A). In addition, patients' sera stimulated PBMCs to produce IL-6, whereas neutralization of PGRN in patients' sera dramatically attenuated such IL-6 production from PBMCs, almost the same as the degradation of DNA in patients' sera (Figure 4B and 4C). IL-6 production in lupus is reported to mediate by immune complexes that transmit signals through TLR7 and 9 [22,32-34] and Fcγ receptor [26]. Our data suggest that DNA-containing immune complexes in patients' sera stimulate IL-6 production from healthy PBMCs, mainly via TLR9 signaling, and that serum PGRN and/or GRN may act as an important cofactor of TLR9 signaling. Conversely, because the augmentation of IL-6 production induced by CpG only in the presence of rhPGRN was limited, it is suggested that cofactors other than PGRN may be required to form immune complexes that effectively stimulate TLR9 signaling.

In our analysis, serum PGRN levels were significantly correlated with serum titers of anti-dsDNA and anti-nRNP antibodies. IL-6 plays a role in patients with SLE, especially on the production of immunoglobulin [35-39]. In addition, lupus B lymphocytes are hypersensitive to IL-6 [40,41]. Thus, induction of IL-6 by PGRN and/or GRN could be a mechanism by which PGRN is correlated with the production of these autoantibodies. Interestingly, anti-Ro antibody, which is strongly associated with increased interferon-pathway activation in SLE [42], was not associated with serum PGRN levels, indicating that PGRN may be involved in the pathogenesis of SLE, independent of the interferon pathway. In addition, serum PGRN levels were significantly elevated with increasing clinical features. Individually, serum PGRN levels were significantly higher in the SLE patients who developed dominant clinical features, namely anemia (*P *= 0.019, data not shown) and arthritis (*P *= 0.002, data not shown). The elevated IL-6 and enhanced inflammation could accelerate these clinical features. IL-6 impairs iron utilization, leading to anemia of chronic disease. In addition, IL-6 promotes arthritis in patients with SLE [37]. Thus, the induction of IL-6 via PGRN is a possible explanation for the fact that PGRN levels are correlated with anemia and arthritis.

We must acknowledge some limitations of this study. The sample size of our study was small. Statistical tests usually require a larger sample size to justify that the effect did not happen by chance alone. Moreover, because of its cross-sectional design, it is difficult to establish the exact and definite causal relations, except the association between PGRN and development of SLE from the collected data.

In conclusion, this pilot study demonstrated that the serum PGRN levels were elevated in patients with SLE and were associated with the systemic disease activity. PGRN could be a useful biomarker for disease activity and may be involved in the pathogenesis of SLE, partly by enhancing the TLR9 signaling. Further studies are required to reveal more-precise mechanisms of PGRN and GRN in human autoimmune diseases and in host defense against microbes.

## Conclusions

The present study demonstrated that the serum levels of PGRN, a fragment of which (GRN) is a soluble cofactor for TLR9 signaling, were elevated in patients with SLE and were associated with the systemic disease activity. PGRN may have a role in the pathogenesis of SLE, partly by affecting TLR9 signaling, and could be a useful biomarker for disease activity. These findings provide new insights into the pathogenesis as well as the therapy of SLE, and shed new light on the dysregulation of the immune system in autoimmune diseases.

## Abbreviations

ACR: American College of Rheumatology; anti-CL: anti-cardiolipin; anti-nRNP: anti-ribonucleoprotein; anti-PGRN Ab: antibody for PGRN; anti-Ro: anti-Ro/SS-A; control Ab: isotype control antibody; CpG-ODN: CpG oligonucleotide; ELISA: enzyme-linked immunosorbent assay; GRN: granulin; PBMCs: peripheral blood mononuclear cells; PBS: phosphate-buffered saline; pDC: plasmacytoid dendritic cell; PGRN: progranulin; PR3: proteinase 3; RA: rheumatoid arthritis; rhPGRN: recombinant human PGRN; SDS-PAGE: sodium dodecyl sulfate-polyacrylamide gel electrophoresis; SLE: systemic lupus erythematosus; SLEDAI: SLE Disease Activity Index; anti-Sm: anti-Smith; TLR: toll-like receptor.

## Competing interests

The authors declare that they have no competing interests.

## Authors' contributions

AT performed the experiments, the statistical analysis, and prepared the manuscript. HT, HM, TH and KA designed the study and helped to draft the manuscript. CK helped the statistical analysis and the manuscript edit. NU, MA, and SO assisted in conducting the experiments. YI, YA and HN contributed to data analysis and interpretation. All authors read and approved the final manuscript.
